# Dichlorido(4′-phenyl-2,2′:6′,2′′-ter­pyridyl)zinc

**DOI:** 10.1107/S1600536812004862

**Published:** 2012-02-17

**Authors:** Zhen Ma, Baohuan Liang, Mei Yang, Lingjun Lu

**Affiliations:** a Guangxi Key Laboratory of Petrochemical Resource Processing and Process Intensification Technology, School of Chemistry and Chemical Engineering, Guangxi University, Nanning, Guangxi 530004, People’s Republic of China

## Abstract

The title compound, [ZnCl_2_(C_21_H_15_N_3_)], was obtained from the reaction of ZnCl_2_·4H_2_O with 4′-phenyl­terpyridine (*L*) and disodium 2,6-dipicolinate. The Zn^2+^ cation is ligated by the N atoms of the tridentate *L* ligand and two chloride anions, forming a ZnN_3_Cl_2_ polyhedron with a distorted trigonal–bipyramidal coordination geometry. In the crystal, nonclassical C—H⋯Cl hydrogen bonds are observed.

## Related literature

For the structures, properties and applications of *MLX*_2_ compounds (*M* = transition metal, *L* = terpyridine, *X* = halogen), see: Bugarcic *et al.* (2004 ▶); Koo *et al.* (2003 ▶); Ma, Liu *et al.* (2009 ▶); Ma, Xing *et al.* (2009 ▶); Ma, Bi *et al.* (2010 ▶); Ma, Cao *et al.* (2010 ▶); Tu *et al.* (2004 ▶); Yam *et al.* (2003 ▶). For the preparation of the ligand, see: Constable *et al.* (1990 ▶). For standard bond lengths, see: Allen *et al.* (1987 ▶).
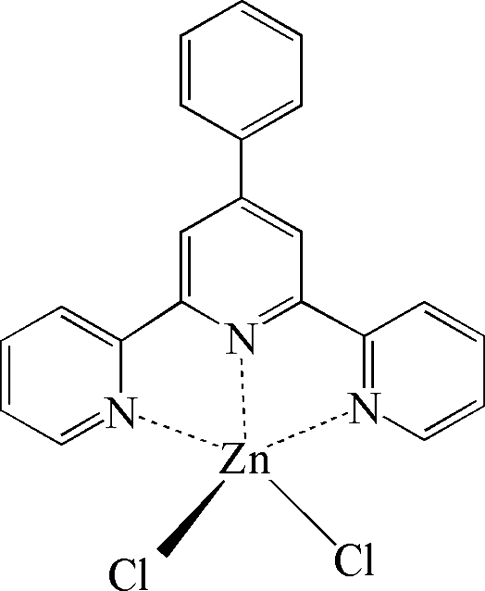


## Experimental

### 

#### Crystal data

[ZnCl_2_(C_21_H_15_N_3_)]
*M* *_r_* = 445.63
Monoclinic, 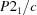

*a* = 12.0728 (10) Å
*b* = 9.5640 (8) Å
*c* = 17.5822 (13) Å
β = 111.386 (5)°
*V* = 1890.3 (3) Å^3^
*Z* = 4
Mo *K*α radiation
μ = 1.59 mm^−1^
*T* = 150 K
0.41 × 0.32 × 0.27 mm

#### Data collection

Bruker SMART CCD area-detector diffractometer
Absorption correction: multi-scan (*SADABS*; Sheldrick, 1996 ▶) *T*_min_ = 0.548, *T*_max_ = 0.651
23122 measured reflections
4711 independent reflections
3809 reflections with *I* > 2σ(*I*)
*R*_int_ = 0.024

#### Refinement

*R*[*F* ^2^ > 2σ(*F* ^2^)] = 0.026
*wR*(*F* ^2^) = 0.066
*S* = 1.01
4711 reflections
244 parameters
H-atom parameters constrained
Δρ_max_ = 0.30 e Å^−3^
Δρ_min_ = −0.24 e Å^−3^

### 

Data collection: *SMART* (Bruker, 2001 ▶); cell refinement: *SAINT* (Bruker, 2002 ▶); data reduction: *SAINT*; program(s) used to solve structure: *SHELXS97* (Sheldrick, 2008 ▶); program(s) used to refine structure: *SHELXL97* (Sheldrick, 2008 ▶); molecular graphics: *SHELXTL* (Sheldrick, 2008 ▶); software used to prepare material for publication: *SHELXL97*.

## Supplementary Material

Crystal structure: contains datablock(s) I, global. DOI: 10.1107/S1600536812004862/wm2584sup1.cif

Structure factors: contains datablock(s) I. DOI: 10.1107/S1600536812004862/wm2584Isup2.hkl

Additional supplementary materials:  crystallographic information; 3D view; checkCIF report

## Figures and Tables

**Table 1:** Selected bond lengths (Å)

Zn1—N2	2.0987 (13)
Zn1—N3	2.1979 (15)
Zn1—N1	2.2000 (15)
Zn1—Cl1	2.2596 (5)
Zn1—Cl2	2.2609 (5)

**Table 2:** Hydrogen-bond geometry (Å, °)

*D*—H⋯*A*	*D*—H	H⋯*A*	*D*⋯*A*	*D*—H⋯*A*
C7—H7*A*⋯Cl1^i^	0.93	2.78	3.546 (2)	140
C12—H12*A*⋯Cl2^ii^	0.93	2.83	3.583 (2)	139
C13—H13*A*⋯Cl2^iii^	0.93	2.83	3.694 (2)	155

Symmetry codes: (i) 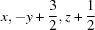; (ii) 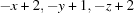; (iii) 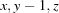.

**Fig. 1.**
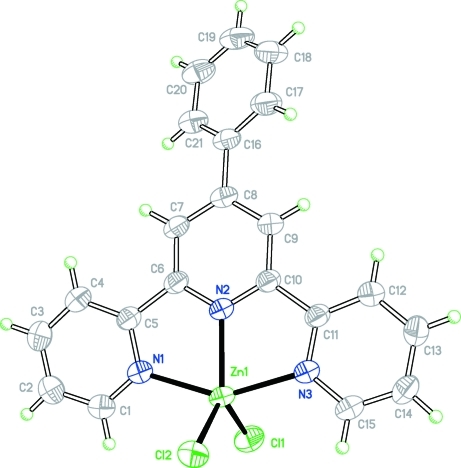
A view of the title complex, showing the atom labelling scheme with 50 % probability displacement ellipsoids. H atoms are presented as small spheres of arbitrary radius. Zn-ligand bonds are indicated by full lines.

**Fig. 2.**
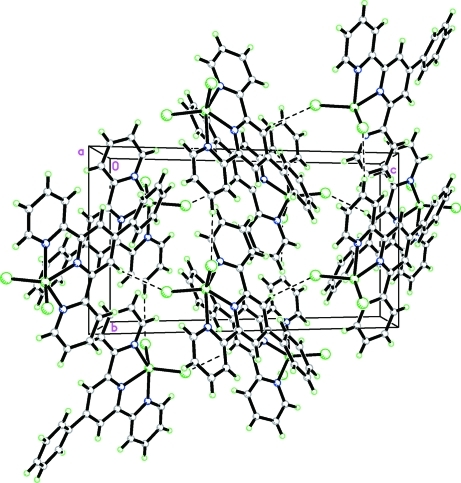
A view of the crystal packing along the a axis. Thin dashed lines are used to show the C—H···Cl hydrogen bonds.

## supplementary crystallographic information

### Comment

This paper forms part of our continuing studies of the synthesis and structural characterization of metal 4'-Ph-terpyridine compounds (Ma, Liu *et al.* (2009); Ma, Xing *et al.* (2009); Ma, Bi *et al.* (2010); Ma, Cao *et al.* (2010)). We are particularly interested in the design and synthesis of metal coordination compounds bearing terpy ligands due to their different coordination topologies and their potential applications in photo-luminescence and antitumor activities. Previous studies on such terpyridine complexes have been published, including Pd(II), Pt(II), Zn(II) and Ag(I) (Bugarcic *et al.*, 2004; Koo *et al.*, 2003; Yam *et al.*, 2003). We report here on the synthesis and the results of the crystal structure analysis of an adduct of zinc chloride with 4'-Ph-terpyridine.

The structure of the title compound, [ZnCl_2_(C_21_H_15_N_3_)], consists of a neutral molecular unit where the metal is penta-coordinate within a [ZnCl_2_*L*] (*L* = 4'-phenyl-2,2':6',2"-terpyridine) (Fig. 1) coordination set. All bond lengths and angles are within normal ranges (Allen *et al.*, 1987). The Zn^2+^ cation is surrounded by the three nitrogen atoms of the ligand and two chloride anions, forming an irregular distorted trigonal-bipyramidal ZnN_3_Cl_2_ polyhedron, whereby the two chloride ions occupy the axial positions and the three equatorial sites are occupied by the nitrogen atoms of *L*. The angles between the apical chloride ions and the three terpy nitrogen atoms range from 96.70 (4) - 123.87 (4) °. The terpyridyl molecule is nearly planar (with an RMS deviations of 0.1029 Å), but the pendant phenyl ring is twisted and makes an angle of 26.55 (9) ° with the plane defined by N1, N2, N3 and Zn1.

No classic hydrogen bonding is observed, but three weak C—H···Cl hydrogen bonds are recognized (Table 2, Fig. 2). The molecules also show an intermolecular C—H···π interaction between a –CH_2_-(C20) and a neighboring five membered group [H···*Cg*^ii^ 2.960 Å, *Cg* is the centroid of the five-membered ring Zn(1)-N(1)-C(5)-C(6)-N(2); symmetry code: (ii) -1-*x*, -*y*, -1-*z*]. Both the hydrogen bonds and the intermolecular interaction help to consolidate the three-dimensional network.

In the title complex no solvent molecule is contained in the structure. However, two crystal structures of [ZnCl_2_*L*] with different solvents (water or dimethylformamide) were already reported (Tu *et al.*, 2004; Ma, Cao *et al.*, 2010).

### Experimental

Free *L* was prepared by a reported procedure (Constable *et al.*, 1990).

The title compound was synthesized by reaction of zinc(II) chloride, *L* and disodium 2,6-dipicolinate (Na_2_C_7_H_3_N_1_O_4_) in the conditions as follows: ZnCl_2_.4H_2_O (0.021 g, 0.10 m*M*), *L* (0.031 g, 0.10 m*M*) were dissoved in a mixture of methanol and DMF (16 cm^3^, 1:1) and a aqueous solution of disodium 2,6-dipicolinate (5 cm^3^, 0.01 M/L) was added. The system was stirred for 48 h at 437 K and cooled down to room temperature. After filtration, a unknown solid and a colorless solution were obtained. Evaporation of the solution gave colorless crystals, which were isolated by mechanical separation from a mixture including an unidentified powder and were suitable for X-ray characterization. The yield of the compound is 16 % (7.3 mg) based on the ligand.

### Refinement

Hydrogen atoms bonded to the ligands were positioned geometrically and refined using a riding model with C—H = 0.93 Å and with Uiso(H) = 1.2×Ueq(C). These hydrogen atoms were assigned isotropic thermal parameters and allowed to ride on their respective parent atoms.

### Crystal data

**Table:**

[ZnCl_2_(C_21_H_15_N_3_)]	*F*(000) = 904
*M**_r_* = 445.63	*D*_x_ = 1.566 Mg m^−^^3^
Monoclinic, *P*2_1_/*c*	Mo *K*α radiation, λ = 0.71073 Å
Hall symbol: -P 2ybc	Cell parameters from 23122 reflections
*a* = 12.0728 (10) Å	θ = 2.5–28.3°
*b* = 9.5640 (8) Å	µ = 1.59 mm^−^^1^
*c* = 17.5822 (13) Å	*T* = 150 K
β = 111.386 (5)°	Prism, colorless
*V* = 1890.3 (3) Å^3^	0.41 × 0.32 × 0.27 mm
*Z* = 4	

### Data collection

**Table:**

Bruker SMART CCD area-detector diffractometer	4711 independent reflections
Radiation source: fine-focus sealed tube	3809 reflections with *I* > 2σ(*I*)
Graphite Monochromator monochromator	*R*_int_ = 0.024
φ and ω scans	θ_max_ = 28.3°, θ_min_ = 2.5°
Absorption correction: multi-scan (*SADABS*; Sheldrick, 1996)	*h* = −14→16
*T*_min_ = 0.548, *T*_max_ = 0.651	*k* = −12→12
23122 measured reflections	*l* = −23→23

### Refinement

**Table:**

Refinement on *F*^2^	Primary atom site location: structure-invariant direct methods
Least-squares matrix: full	Secondary atom site location: difference Fourier map
*R*[*F*^2^ > 2σ(*F*^2^)] = 0.026	Hydrogen site location: inferred from neighbouring sites
*wR*(*F*^2^) = 0.066	H-atom parameters constrained
*S* = 1.01	*w* = 1/[σ^2^(*F*_o_^2^) + (0.0261*P*)^2^ + 0.8758*P*] where *P* = (*F*_o_^2^ + 2*F*_c_^2^)/3
4711 reflections	(Δ/σ)_max_ = 0.001
244 parameters	Δρ_max_ = 0.30 e Å^−^^3^
0 restraints	Δρ_min_ = −0.24 e Å^−^^3^

### Special details

**Table:**

Geometry. All esds (except the esd in the dihedral angle between two l.s. planes) are estimated using the full covariance matrix. The cell esds are taken into account individually in the estimation of esds in distances, angles and torsion angles; correlations between esds in cell parameters are only used when they are defined by crystal symmetry. An approximate (isotropic) treatment of cell esds is used for estimating esds involving l.s. planes.
Refinement. Refinement of F^2^ against ALL reflections. The weighted R-factor wR and goodness of fit S are based on F^2^, conventional R-factors R are based on F, with F set to zero for negative F^2^. The threshold expression of F^2^ > 2sigma(F^2^) is used only for calculating R-factors(gt) etc. and is not relevant to the choice of reflections for refinement. R-factors based on F^2^ are statistically about twice as large as those based on F, and R- factors based on ALL data will be even larger.

### Fractional atomic coordinates and isotropic or equivalent isotropic displacement parameters (Å^2^)

**Table:**

	*x*	*y*	*z*	*U*_iso_*/*U*_eq_	
Zn1	0.826519 (19)	0.72657 (2)	0.855588 (11)	0.04016 (7)	
Cl1	0.69654 (5)	0.70715 (6)	0.72558 (3)	0.05675 (13)	
Cl2	0.99013 (4)	0.85910 (5)	0.87665 (3)	0.05130 (12)	
N1	0.72851 (13)	0.87880 (16)	0.90086 (8)	0.0417 (3)	
N2	0.78944 (12)	0.62385 (15)	0.94908 (8)	0.0363 (3)	
N3	0.90559 (13)	0.51746 (15)	0.86385 (8)	0.0408 (3)	
C1	0.70079 (18)	1.0079 (2)	0.87212 (12)	0.0503 (4)	
H1A	0.7176	1.0349	0.8267	0.060*	
C2	0.64816 (19)	1.1033 (2)	0.90694 (13)	0.0556 (5)	
H2A	0.6262	1.1914	0.8840	0.067*	
C3	0.62875 (19)	1.0653 (2)	0.97635 (13)	0.0559 (5)	
H3A	0.5961	1.1291	1.0023	0.067*	
C4	0.65785 (18)	0.9324 (2)	1.00747 (11)	0.0492 (4)	
H4A	0.6461	0.9054	1.0548	0.059*	
C5	0.70490 (15)	0.83993 (19)	0.96679 (10)	0.0385 (4)	
C6	0.73050 (15)	0.69077 (19)	0.98985 (9)	0.0372 (4)	
C7	0.69419 (16)	0.62213 (19)	1.04596 (9)	0.0403 (4)	
H7A	0.6542	0.6707	1.0740	0.048*	
C8	0.71776 (15)	0.48016 (19)	1.06039 (9)	0.0390 (4)	
C9	0.78203 (16)	0.41385 (19)	1.01902 (10)	0.0404 (4)	
H9A	0.8017	0.3198	1.0285	0.048*	
C10	0.81661 (15)	0.48883 (18)	0.96363 (9)	0.0362 (3)	
C11	0.88390 (15)	0.42766 (18)	0.91567 (9)	0.0367 (3)	
C12	0.92301 (17)	0.29096 (19)	0.92334 (11)	0.0445 (4)	
H12A	0.9066	0.2303	0.9592	0.053*	
C13	0.98702 (18)	0.2453 (2)	0.87693 (12)	0.0496 (4)	
H13A	1.0140	0.1535	0.8811	0.060*	
C14	1.01030 (18)	0.3371 (2)	0.82457 (11)	0.0498 (4)	
H14A	1.0539	0.3091	0.7932	0.060*	
C15	0.96758 (18)	0.4716 (2)	0.81954 (11)	0.0487 (4)	
H15A	0.9826	0.5334	0.7836	0.058*	
C16	0.67254 (16)	0.3996 (2)	1.11540 (10)	0.0415 (4)	
C17	0.7271 (2)	0.2763 (2)	1.15197 (12)	0.0533 (5)	
H17A	0.7972	0.2475	1.1462	0.064*	
C18	0.6775 (2)	0.1954 (2)	1.19729 (12)	0.0611 (6)	
H18A	0.7137	0.1121	1.2208	0.073*	
C19	0.5750 (2)	0.2383 (2)	1.20741 (12)	0.0590 (6)	
H19A	0.5412	0.1831	1.2367	0.071*	
C20	0.52267 (18)	0.3626 (3)	1.17430 (12)	0.0563 (5)	
H20A	0.4551	0.3932	1.1829	0.068*	
C21	0.57041 (17)	0.4428 (2)	1.12796 (11)	0.0487 (4)	
H21A	0.5338	0.5263	1.1051	0.058*	

### Atomic displacement parameters (Å^2^)

**Table:**

	*U*^11^	*U*^22^	*U*^33^	*U*^12^	*U*^13^	*U*^23^
Zn1	0.05320 (13)	0.04105 (12)	0.03372 (10)	−0.00062 (9)	0.02474 (9)	0.00281 (8)
Cl1	0.0724 (3)	0.0640 (3)	0.0343 (2)	0.0018 (2)	0.0199 (2)	0.0009 (2)
Cl2	0.0591 (3)	0.0462 (3)	0.0567 (3)	−0.0048 (2)	0.0307 (2)	0.0041 (2)
N1	0.0520 (9)	0.0415 (8)	0.0373 (7)	0.0009 (6)	0.0229 (6)	0.0018 (6)
N2	0.0439 (8)	0.0386 (8)	0.0305 (6)	0.0000 (6)	0.0187 (6)	0.0006 (5)
N3	0.0531 (9)	0.0403 (8)	0.0361 (7)	0.0008 (6)	0.0245 (6)	0.0013 (6)
C1	0.0650 (12)	0.0455 (11)	0.0466 (10)	0.0017 (9)	0.0276 (9)	0.0056 (8)
C2	0.0685 (13)	0.0421 (11)	0.0596 (12)	0.0074 (9)	0.0273 (10)	0.0049 (9)
C3	0.0669 (13)	0.0475 (12)	0.0613 (12)	0.0076 (10)	0.0328 (10)	−0.0058 (9)
C4	0.0605 (12)	0.0507 (11)	0.0454 (9)	0.0026 (9)	0.0301 (9)	−0.0023 (8)
C5	0.0430 (9)	0.0414 (9)	0.0338 (8)	−0.0008 (7)	0.0173 (7)	−0.0017 (7)
C6	0.0415 (9)	0.0421 (9)	0.0306 (7)	−0.0013 (7)	0.0163 (7)	−0.0022 (6)
C7	0.0462 (9)	0.0480 (10)	0.0317 (7)	−0.0009 (8)	0.0203 (7)	−0.0020 (7)
C8	0.0413 (9)	0.0492 (10)	0.0285 (7)	−0.0040 (7)	0.0148 (7)	0.0017 (7)
C9	0.0494 (10)	0.0402 (9)	0.0360 (8)	0.0000 (7)	0.0209 (7)	0.0043 (7)
C10	0.0409 (9)	0.0395 (9)	0.0303 (7)	−0.0012 (7)	0.0154 (6)	−0.0009 (6)
C11	0.0423 (9)	0.0405 (9)	0.0299 (7)	−0.0014 (7)	0.0162 (6)	−0.0016 (6)
C12	0.0553 (11)	0.0422 (10)	0.0395 (9)	0.0012 (8)	0.0216 (8)	0.0025 (7)
C13	0.0606 (12)	0.0424 (11)	0.0488 (10)	0.0062 (8)	0.0234 (9)	−0.0046 (8)
C14	0.0597 (12)	0.0547 (12)	0.0428 (9)	0.0045 (9)	0.0280 (9)	−0.0070 (8)
C15	0.0619 (12)	0.0521 (11)	0.0436 (9)	−0.0001 (9)	0.0329 (9)	0.0006 (8)
C16	0.0469 (10)	0.0504 (10)	0.0303 (7)	−0.0068 (8)	0.0175 (7)	0.0014 (7)
C17	0.0611 (12)	0.0607 (13)	0.0450 (10)	0.0034 (10)	0.0276 (9)	0.0107 (9)
C18	0.0788 (15)	0.0610 (14)	0.0476 (11)	−0.0018 (11)	0.0280 (10)	0.0156 (10)
C19	0.0674 (13)	0.0727 (15)	0.0407 (10)	−0.0248 (11)	0.0242 (9)	0.0033 (9)
C20	0.0488 (11)	0.0796 (16)	0.0468 (10)	−0.0141 (10)	0.0251 (9)	−0.0001 (10)
C21	0.0493 (10)	0.0593 (12)	0.0409 (9)	−0.0050 (9)	0.0203 (8)	0.0035 (8)

### Geometric parameters (Å, º)

**Table:**

Zn1—N2	2.0987 (13)	C8—C16	1.488 (2)
Zn1—N3	2.1979 (15)	C9—C10	1.390 (2)
Zn1—N1	2.2000 (15)	C9—H9A	0.9300
Zn1—Cl1	2.2596 (5)	C10—C11	1.488 (2)
Zn1—Cl2	2.2609 (5)	C11—C12	1.380 (3)
N1—C1	1.330 (2)	C12—C13	1.383 (3)
N1—C5	1.343 (2)	C12—H12A	0.9300
N2—C10	1.334 (2)	C13—C14	1.374 (3)
N2—C6	1.342 (2)	C13—H13A	0.9300
N3—C15	1.336 (2)	C14—C15	1.376 (3)
N3—C11	1.346 (2)	C14—H14A	0.9300
C1—C2	1.376 (3)	C15—H15A	0.9300
C1—H1A	0.9300	C16—C17	1.389 (3)
C2—C3	1.373 (3)	C16—C21	1.392 (3)
C2—H2A	0.9300	C17—C18	1.393 (3)
C3—C4	1.378 (3)	C17—H17A	0.9300
C3—H3A	0.9300	C18—C19	1.375 (3)
C4—C5	1.383 (2)	C18—H18A	0.9300
C4—H4A	0.9300	C19—C20	1.372 (3)
C5—C6	1.484 (3)	C19—H19A	0.9300
C6—C7	1.383 (2)	C20—C21	1.388 (3)
C7—C8	1.392 (3)	C20—H20A	0.9300
C7—H7A	0.9300	C21—H21A	0.9300
C8—C9	1.395 (2)		
			
N2—Zn1—N3	74.56 (5)	C7—C8—C16	121.66 (16)
N2—Zn1—N1	74.33 (5)	C9—C8—C16	120.63 (16)
N3—Zn1—N1	148.89 (5)	C10—C9—C8	119.80 (16)
N2—Zn1—Cl1	119.08 (4)	C10—C9—H9A	120.1
N3—Zn1—Cl1	96.70 (4)	C8—C9—H9A	120.1
N1—Zn1—Cl1	98.84 (4)	N2—C10—C9	121.20 (15)
N2—Zn1—Cl2	123.87 (4)	N2—C10—C11	114.77 (14)
N3—Zn1—Cl2	99.60 (4)	C9—C10—C11	124.03 (16)
N1—Zn1—Cl2	97.05 (4)	N3—C11—C12	121.80 (15)
Cl1—Zn1—Cl2	117.05 (2)	N3—C11—C10	114.47 (15)
C1—N1—C5	118.79 (16)	C12—C11—C10	123.73 (15)
C1—N1—Zn1	124.82 (12)	C11—C12—C13	119.10 (17)
C5—N1—Zn1	116.14 (12)	C11—C12—H12A	120.4
C10—N2—C6	120.02 (14)	C13—C12—H12A	120.4
C10—N2—Zn1	119.71 (11)	C14—C13—C12	119.22 (18)
C6—N2—Zn1	120.07 (11)	C14—C13—H13A	120.4
C15—N3—C11	118.29 (16)	C12—C13—H13A	120.4
C15—N3—Zn1	125.63 (12)	C13—C14—C15	118.50 (17)
C11—N3—Zn1	116.01 (11)	C13—C14—H14A	120.7
N1—C1—C2	122.63 (18)	C15—C14—H14A	120.7
N1—C1—H1A	118.7	N3—C15—C14	123.09 (17)
C2—C1—H1A	118.7	N3—C15—H15A	118.5
C3—C2—C1	118.47 (19)	C14—C15—H15A	118.5
C3—C2—H2A	120.8	C17—C16—C21	118.48 (17)
C1—C2—H2A	120.8	C17—C16—C8	121.08 (17)
C2—C3—C4	119.69 (19)	C21—C16—C8	120.36 (17)
C2—C3—H3A	120.2	C16—C17—C18	120.4 (2)
C4—C3—H3A	120.2	C16—C17—H17A	119.8
C3—C4—C5	118.55 (18)	C18—C17—H17A	119.8
C3—C4—H4A	120.7	C19—C18—C17	120.2 (2)
C5—C4—H4A	120.7	C19—C18—H18A	119.9
N1—C5—C4	121.73 (17)	C17—C18—H18A	119.9
N1—C5—C6	114.40 (14)	C20—C19—C18	120.05 (19)
C4—C5—C6	123.85 (15)	C20—C19—H19A	120.0
N2—C6—C7	121.49 (16)	C18—C19—H19A	120.0
N2—C6—C5	114.23 (14)	C19—C20—C21	120.2 (2)
C7—C6—C5	124.24 (15)	C19—C20—H20A	119.9
C6—C7—C8	119.76 (16)	C21—C20—H20A	119.9
C6—C7—H7A	120.1	C20—C21—C16	120.67 (19)
C8—C7—H7A	120.1	C20—C21—H21A	119.7
C7—C8—C9	117.67 (15)	C16—C21—H21A	119.7
			
N2—Zn1—N1—C1	−179.81 (17)	N1—C5—C6—C7	−168.21 (16)
N3—Zn1—N1—C1	178.96 (14)	C4—C5—C6—C7	10.2 (3)
Cl1—Zn1—N1—C1	−61.95 (16)	N2—C6—C7—C8	−0.7 (3)
Cl2—Zn1—N1—C1	56.93 (16)	C5—C6—C7—C8	176.87 (16)
N2—Zn1—N1—C5	6.05 (12)	C6—C7—C8—C9	2.4 (2)
N3—Zn1—N1—C5	4.82 (19)	C6—C7—C8—C16	−175.30 (15)
Cl1—Zn1—N1—C5	123.91 (12)	C7—C8—C9—C10	−2.1 (2)
Cl2—Zn1—N1—C5	−117.21 (12)	C16—C8—C9—C10	175.64 (15)
N3—Zn1—N2—C10	−6.33 (12)	C6—N2—C10—C9	1.8 (2)
N1—Zn1—N2—C10	174.33 (14)	Zn1—N2—C10—C9	−173.10 (12)
Cl1—Zn1—N2—C10	82.81 (13)	C6—N2—C10—C11	−178.90 (14)
Cl2—Zn1—N2—C10	−97.65 (12)	Zn1—N2—C10—C11	6.16 (19)
N3—Zn1—N2—C6	178.74 (14)	C8—C9—C10—N2	0.0 (3)
N1—Zn1—N2—C6	−0.59 (12)	C8—C9—C10—C11	−179.19 (15)
Cl1—Zn1—N2—C6	−92.12 (12)	C15—N3—C11—C12	−0.5 (3)
Cl2—Zn1—N2—C6	87.42 (13)	Zn1—N3—C11—C12	176.50 (13)
N2—Zn1—N3—C15	−177.69 (16)	C15—N3—C11—C10	178.75 (15)
N1—Zn1—N3—C15	−176.45 (14)	Zn1—N3—C11—C10	−4.23 (18)
Cl1—Zn1—N3—C15	63.93 (15)	N2—C10—C11—N3	−1.0 (2)
Cl2—Zn1—N3—C15	−55.03 (15)	C9—C10—C11—N3	178.25 (16)
N2—Zn1—N3—C11	5.53 (12)	N2—C10—C11—C12	178.27 (16)
N1—Zn1—N3—C11	6.77 (19)	C9—C10—C11—C12	−2.5 (3)
Cl1—Zn1—N3—C11	−112.85 (12)	N3—C11—C12—C13	0.5 (3)
Cl2—Zn1—N3—C11	128.19 (12)	C10—C11—C12—C13	−178.71 (17)
C5—N1—C1—C2	−0.4 (3)	C11—C12—C13—C14	0.2 (3)
Zn1—N1—C1—C2	−174.39 (16)	C12—C13—C14—C15	−0.7 (3)
N1—C1—C2—C3	3.1 (3)	C11—N3—C15—C14	−0.1 (3)
C1—C2—C3—C4	−2.5 (3)	Zn1—N3—C15—C14	−176.81 (15)
C2—C3—C4—C5	−0.7 (3)	C13—C14—C15—N3	0.7 (3)
C1—N1—C5—C4	−3.0 (3)	C7—C8—C16—C17	−157.38 (18)
Zn1—N1—C5—C4	171.47 (14)	C9—C8—C16—C17	25.0 (3)
C1—N1—C5—C6	175.42 (16)	C7—C8—C16—C21	26.0 (2)
Zn1—N1—C5—C6	−10.07 (19)	C9—C8—C16—C21	−151.68 (18)
C3—C4—C5—N1	3.6 (3)	C21—C16—C17—C18	2.6 (3)
C3—C4—C5—C6	−174.71 (18)	C8—C16—C17—C18	−174.12 (18)
C10—N2—C6—C7	−1.5 (2)	C16—C17—C18—C19	−1.2 (3)
Zn1—N2—C6—C7	173.40 (12)	C17—C18—C19—C20	−1.4 (3)
C10—N2—C6—C5	−179.26 (15)	C18—C19—C20—C21	2.4 (3)
Zn1—N2—C6—C5	−4.35 (19)	C19—C20—C21—C16	−0.9 (3)
N1—C5—C6—N2	9.5 (2)	C17—C16—C21—C20	−1.6 (3)
C4—C5—C6—N2	−172.12 (17)	C8—C16—C21—C20	175.17 (17)

### Hydrogen-bond geometry (Å, º)

**Table:**

*D*—H···*A*	*D*—H	H···*A*	*D*···*A*	*D*—H···*A*
C7—H7*A*···Cl1^i^	0.93	2.78	3.546 (2)	140
C12—H12*A*···Cl2^ii^	0.93	2.83	3.583 (2)	139
C13—H13*A*···Cl2^iii^	0.93	2.83	3.694 (2)	155

Symmetry codes: (i) *x*, −*y*+3/2, *z*+1/2; (ii) −*x*+2, −*y*+1, −*z*+2; (iii) *x*, *y*−1, *z*.
